# Calcitonin Gene-Related Peptide (CGRP) in Cerebrovascular Disease

**DOI:** 10.1100/tsw.2002.806

**Published:** 2002-05-30

**Authors:** Lars Edvinsson

**Affiliations:** Department of Internal Medicine, Lund University Hospital, S-221 85 Lund, Sweden

**Keywords:** migraine, cluster headache, CGRP, subarachnoid hemorrhage, SAH, VIP, trigeminovascular reflex

## Abstract

Cerebral blood vessels are innervated by sensory nerves that store several neurotransmitters among which calcitonin gene-related peptide (CGRP) is the most abundant. In primary headaches, there is a clear association between the head pain and the release of CGRP. In cluster headache there is an additional release of vasoactive intestinal peptide (VIP).

In connection with administration of triptans, the headache subsides and the neuropeptide release normalises, in part via a presynaptic effect.

In subarachnoid hemorrhage (SAH), CGRP is released to counterbalance the blood-induced vasospasm. In severe cases, the stored CGRP may be exhausted while infusion of CGRP may limit cerebral vasospasm. Thus, interactions with the trigeminovascular system at CGRP receptors may be a useful target for understanding of cerebrovascular disease and to design novel treatments.

## INTRODUCTION

Calcitonin gene-related peptide (CGRP) is a 37–amino acid neuropeptide, first identified in 1982[1]. It belongs to a family of peptides, which includes calcitonin, adrenomedullin, and amylin. Localisation studies have shown a wide distribution of CGRP immunoreactivity in the peripheral and in the central nervous systems[2,3]. Although CGRP-containing nerves innervate blood vessels in various regions, and CGRP is a potent vasodilator, it has other effects within the cardiovascular system and is involved in pain. The role of CGRP in the cardiovascular system is not well understood in normal or pathological states[3,4,5].

In one particular region, the cerebral blood vessels, there is a dense supply of CGRP-containing nerve fibres, which originate in the trigeminal ganglion[6,7]. The CGRP-containing cell bodies of the *trigeminal system* are bipolar and have functional connections with neurons in the trigeminal nucleus caudalis and in its related extensions down to the $C1_{1\text{-}2}$ level[8]. The peripheral part innervates intracranial vessels via its ophthalmic division. These fibres release CGRP following electrical stimulation[9] or capsaicin treatment[10,11] and are activated in primary headaches and in stroke[12]. Trigeminal fibres mediate dilatation of brain vessels[13,14], mediate increases in cerebral blood flow[15], and participate in the trigeminovascular reflex[13]. The trigeminovascular system is a vasodilator pathway, which acts via antidromic release of CGRP upon activation[11,13] and has a primary involvement in sensory functions. Although CGRP has a number of effects, its most pronounced action is vasodilatation[5].

Early studies demonstrated strong vasomotor effects of CGRP on a variety of tissues[3,4,5,6]. CGRP is present in two forms, α- and β-CGRP. Calcitonin and α-CGRP are transcribed from the same gene, while β-CGRP is formed from a duplication of the calcitonin αCGRP gene. Early pharmacological studies focused on the use of CGRP agonists to discriminate between CGRP receptor subtypes. The $CGRP_1$ receptor was characterised as being particularly sensitive to antagonism by the CGRP fragment $CGRP_{8\text{-}37}$, with reported $pA_2$ values in the range of 1–100 nM, while the $CGRP_2$ receptors had lower affinity for this blocker, often in the μM region.

$CGRP_{8\text{-}37}$ has no agonist activity at concentrations up to 10 μM. A third class of CGRP binding sites has been identified in the rat nucleus accumbens that recognises both α-CGRP and calcitonin with high affinity[16]. Subsequent cloning efforts have resulted in the molecular identification of the $CGRP_1$ receptor, calcitonin receptor–like receptor (CRLR)[17]. CRLR is a $G_s$-coupled seven-transmembrane domain receptor (GPCR), which shares 55% sequence identity with the calcitonin receptor. McLatchie and coworkers[18] demonstrated that functional $CGRP_1$ and adrenomedullin receptors are both derived from CRLR and that the phenotype is determined by coexpression with a particular receptor activity modifying protein, RAMP. Coexpression of CRLR with RAMP1 results in $CGRP_1$ receptor pharmacology while RAMP2 or RAMP3 coexpression produces an adrenomedullin receptor. RAMPs are relatively small (148–175 amino acids) proteins containing a single-membrane spanning domain, a large extracellular domain, and a short cytoplasmic domain. The RAMPs enable expression of CRLR on the cell surface, determine the glycosylation state of the receptor, and determine the relative affinity of this receptor for CGRP and adrenomedullin[19]. CRLR internalisation following CGRP stimulation has been shown to occur together with RAMP1, and both proteins are targeted to the protein degradation pathway[20]. Although the receptors resulting from coexpression of CRLR with either RAMP1 or RAMP2/3 are functionally distinct and glycosylated to different extents, CGRP and adrenomedullin display some cross-reactivity for the opposite receptor[18]. RAMP proteins have, in addition, been shown to modulate the pharmacology of the calcitonin receptor, which in combination with RAMP1 or RAMP3, binds amylin with high affinity[21,22]. In addition to the RAMPs, the CGRP receptor may require another accessory protein for function, the receptor component protein or RCP[23].

## THE TRIGEMINOVASCULAR REFLEX

Following the identification of the trigeminal vascular pathway and its messenger molecules[8], functional studies were initiated, and it was observed that denervation did not alter the regional cerebral blood flow or the regional cerebral metabolism, the cerebral vascular responses to carbon dioxide, or the cerebral autoregulation[24]. However, vasoconstrictor responses elicited by noradrenaline[13], alkaline pH, $PGF_{2\alpha}$, $BaCl_2$, and subarachnoid blood[24,25] were modified. Following denervation, there was no alteration in the contractile response to the listed agents, but the time to attain initial basal tone was markedly prolonged. Vasoconstriction triggers an antidromic release of sensory neuropeptides, e.g., CGRP, which normalises the vessel tone. Subsequent studies using neuropeptide receptor antagonists and capsaicin in combination with denervation showed that it is CGRP that is mainly responsible for this response[11,25]. Vasodilation of cortical arterioles induced by acidic pH was not modified by trigeminal lesion. Thus, if the headache or a vasospasm is initiated by a spreading wave of depression of cortical neurons and/or intense vasoconstriction, the trigeminal vascular system may have a counter-balancing effect, resulting in a normalised vascular tone (by the release of CGRP). The activation of this system is noted clinically as an increase in the cranial venous outflow of CGRP during headache attacks or in subarachnoid haemorrhage where it was elevated also in the CSF (see below).

## NEUROTRANSMITTER RELEASE IN PRIMARY HEADACHES

With a better knowledge and understanding of the organisation of the sensory nerves around the intracranial vessels[26], we have studied their involvement in primary headaches by analysis of neurotransmitter release into the cranial venous outflow[12]. In humans, stimulation of the trigeminal ganglion results in unilateral blood flow increases. In trigeminal neuralgia patients, there is, in addition, flushing on the side of stimulation, which coincides with the release of CGRP and substance P[9].

In migraine attacks, there are both vascular and neurogenic components[12]. At present, many believe the disorder is due to mutation of a calcium channel gene rendering some central nervous system neurons to be unstable and capable of initiating a migraine attack. As part of the pathophysiological mechanisms, the trigeminovascular system is activated (putatively as a defense mechanism), and there is release of CGRP and activation of the trigeminal nucleus caudalis (TNC) with subsequent mediation of pain. The results from trigeminal ganglion stimulation in trigeminal neuralgia patients (neuropeptides are released and can be measured in the jugular vein) led us to examine the levels of various neuropeptides during migraine attacks. The concentrations of neuropeptide Y (NPY marker for the sympathetic nerves), vasoactive intestinal polypeptide (VIP, parasympathetic activity), and CGRP and substance P (markers for sensory nerves) were measured. There were no changes in the levels of the peptides NPY, VIP or substance P in the jugular vein plasma. However, a marked increase in CGRP was observed during migraine headache[27]. Two individuals with facial symptoms similar to those seen in cluster headache (e.g., nasal congestion and rhinorrhea) displayed increases in VIP. This suggests the involvement of a parasympathetically mediated event in these two individuals. There was no difference between migraine with aura or without aura.

We propose that the release of CGRP is due to the fact that the cerebral circulation is preferentially innervated by CGRP-containing fibres from the trigeminal ganglion[28]. These observations have been confirmed in subsequent studies[29,30]. In addition, following sumatriptan administration, the plasma levels of CGRP returned to control with successful amelioration of the headache[30].

Cluster headache is a well-described, clear-cut, clinical syndrome. Patients with episodic cluster headache, fulfilling the criteria of the International Headache Society, were examined during acute spontaneous attacks of headache to determine the local cranial release of neuropeptides[31]. During the attacks, the blood levels of CGRP and VIP were markedly raised, while there was no changes in the levels of NPY or substance P. Treatment with oxygen or subcutaneous sumatriptan reduced the CGRP levels to normal, while opiate administration did not alter the peptide levels[31]. The results show that activation of the trigeminovascular system and the cranial parasympathetic nervous system have a role in acute attacks of cluster headache. It was particularly noteworthy that all subjects responded with release of VIP. This is in concert with the facial symptoms in this disorder. Furthermore, it was also shown that both oxygen and sumatriptan, while aborting the attacks, terminated the activity in the trigeminovascular system. This agrees well with the results of others[32,33] demonstrating the release of CGRP in nitroglycerine-elicited attacks of cluster headache. CGRP in the jugular vein on the pain side in cluster-headache patients was found to be elevated during the attack period and was elevated further at the peak of the provoked attack. There were no alterations in substance P levels. Interestingly, only when the subjects were in an active period was nitroglycerine able to elicit an attack of cluster headache[33]. This suggests that the trigeminovascular system is hyperreactive at this stage.

Thus, CGRP, a marker of the trigeminovascular system, and VIP, a marker of the parasympathetic nerve activity, are both elevated in the cranial venous blood of patients suffering acute spontaneous attacks of cluster headache. The termination of the attack with either sumatriptan or oxygen normalised the CGRP levels, probably reflecting cessation of the activity in the trigeminovascular system. In contrast, administration of an opiate agonist induced pain relief, but did not end the trigeminovascular activity. The finding of elevated levels of both CGRP and VIP in the cranial venous blood during attacks suggests that there is activation of a brainstem reflex, the afferent arc of which is the trigeminal nerve and the efferent the cranial parasympathetic outflow from the VIIth nerve.

## SUBARACHNOID HEMORRHAGE

Strong support for the involvement of this reflex in cerebrovascular disorders has come from studies of subarachnoid haemorrhage (SAH), both in patients and in laboratory animals. The first study on the involvement of perivascular sensory fibres containing substance P and CGRP in conjunction with subarachnoid hemorrhage (SAH) showed 2 days after blood injection, the time point at which maximum vasoconstriction is occurring in this model, a reduction in substance P and CGRP in the vessel walls immunoreactivity[34]. The quantitative measurements revealed a 50% reduction of CGRP and only in a slight reduction of substance P in SAH as compared to controls. This partial reduction in neurotransmitter content caused no change in the sensitivity of the basilar artery to substance P or CGRP. However, the maximum relaxant response to CGRP was increased from 52 to 81% ($p < 0.05$), while there was no change in the maximum substance P-induced relaxations. It is suggested that not only a pre-, but also a postsynaptic modulation of perivascular sensory fibres may occur in experimental SAH[34].

This was verified in a study on patients who died from SAH[35]. Their cerebral vessels were depleted of CGRP, but not of other neuronal messengers, indicating a selective loss of the sensory nerve transmitter CGRP that is putatively used to counterbalance the haemorrhage-induced vasoconstriction.

In order to further learn about the possible significance of the trigeminovascular system, we have examined patients that have been admitted to the clinic due to an acute SAH[36,37]. After operation with aneurysm clipping and treatment with the calcium channel blocker nimodipine, the degree of vasoconstriction was monitored with Doppler ultrasound recordings bilaterally from the middle cerebral and internal carotid arteries. External jugular vein blood sampling for neuropeptides analysis was analysed every second day. The highest CGRP levels were found in patients with the highest velocity index values (vasospasm). In patients with middle cerebral artery (MCA) aneurysms, a significant correlation was found between the vasospasm index and the CGRP levels. There were no changes observed in the substance P or VIP levels. Alterations in cerebrovascular tone and in CBF, induced by changing arterial $CO_2$ tension or by lowering of blood pressure (autoregulation test), did not alter the levels of the perivascular peptides[37]. In individual patients with marked vasoconstriction, due to SAH, increased levels of CGRP, and NPY were also observed in the cerebrospinal fluid (CSF), thus, complementing the venous outflow peptide data. These results demonstrate that, in a strict and clearcut intracranial arterial vasoconstrictory disorder, the trigeminovascular reflex is activated to counterbalance vasospasm by release of the vasodilator CGRP. This is verified both by measurements in the jugular vein outflow and in the CSF. In addition, subjects that had vasospasm and cerebral ischemia were treated with CGRP infusion[38,39]. A multicenter study[39] could not show a significant beneficial effect of CGRP in this trial. The wide confidence interval for the risk of a poor outcome and the fact that only a third of the patients completed treatment mean that a clinically useful benefit could not be ruled out. Since few patients completed the study in part because of hypotension, we continued with another CGRP infusion study and careful monitoring[38]. The study was designed to evaluate the effects of intravenous administration of human α-CGRP on cerebral vasoconstriction in the postoperative course after SAH[38]. Cerebral vasoconstriction was evaluated with transcranial Dopler sonography. The increase in the relationship between middle cerebral artery (MCA) velocity and internal carotid artery (ICA) velocity (the hemodynamic index) was used as an indicator of vasoconstriction and compared to the contralateral side. A significant reduction was found in the hemodynamic index during the CGRP infusion ($4.3 \pm 0.5$, $p < 0.05$) as compared to before infusion ($6.2 \pm 0.5$). There was no measurable change in the hemodynamic index on the contralateral side. No significant change was observed in pulsatility index, blood pressure, or consciousness during the peptide infusion. The results obtained show that infusion of human α-CGRP may induce normalisation of cerebrovascular tone in SAH. Another way to increase the availability of CGRP to cerebral vessels in SAH is by gene transfer. This was found to be successful in the rabbit[40], proving that the CGRP approach may be beneficial in cerebrovascular disease.

## CONCLUSION

CGRP seems to have a significant role in cerebrovascular disease. Adding the peptide or a specific molecule with agonistic effect may prove of value in SAH with vasospasm and in stroke. In primary headaches the novel CGRP antagonists have in clinical trials proven that they can ameliorate the headache and thus prove the concept. The development of further molecules in this field is anticipated.
